# Hydrogen production from formic acid catalyzed by NHC–Cu complexes

**DOI:** 10.3762/bjoc.22.48

**Published:** 2026-04-23

**Authors:** Orlando Santoro, Catherine S J Cazin

**Affiliations:** 1 EaStCHEM School of Chemistry, University of St Andrews, St Andrews, KY16 9ST, UK; 2 Dipartimento di Biotecnologie e Scienze della Vita, Università degli Studi dell’Insubria, Via Dunant 3, 21100 Varese, Italy; 3 Department of Chemistry, Ghent University, Krijgslaan 289, 9000 Gent, Belgium

**Keywords:** copper N-heterocyclic carbene, formic acid dehydrogenation, hydrogen storage, metal hydride, silanes

## Abstract

The first NHC–Cu-catalyzed decomposition of formic acid (FA) is reported. In the presence of PhSiH_3_, only hydrogen is generated while CO_2_ is captured by a silane species. The decomposition of an equimolar mixture of FA and an amine provided an equimolar mixture of H_2_ and CO_2_. The efficiency of the catalysis showed to be strongly dependent on the nature of the amine.

## Introduction

The discovery and utilization of alternative and sustainable energy sources is nowadays one of biggest challenges faced by the scientific community and society. Because of its high energy content, hydrogen has been proposed as a possible solution [1–5]. However, the problems connected to its generation, delivery, and storage are limitations to the realization of a hydrogen economy [6–7]. Amongst the various H_2_ sources investigated to date, formic acid (HCO_2_H, FA) is considered one of the most promising. Indeed, FA contains 4.4% H_2_, is inexpensive and is liquid under ambient conditions, thus easy to handle and transport. In addition, the CO_2_ generated from its dehydrogenation could be recycled by hydrogenation to methanol or formic acid [8–10].

The catalytic FA dehydrogenation has been mediated using several transition metals [11–14]. In this context, noble metals such as Ru [15–23], Ir [24–29] and Rh [30–31] have shown to allow the selective transformation of formic acid into H_2_ and CO_2_ with high turnover frequencies (TOFs). In most cases, addition of a base (either amines or formate salts) is required to obtain high catalytic activity. Efficient systems based on inexpensive metals have also been explored. Beller and co-workers reported the first Fe catalyst bearing a phosphine ligand able to produce H_2_ with a TON of 1942 in the absence of additives [32]. A well-defined pincer–Fe complex allowing FA decomposition was reported by Milstein and co-workers [33]. While high TONs (up to 100,000) were achieved in the presence of a trialkylamine, no conversion was observed in the absence of additives. Other Fe-based systems proving highly performing in the FA dehydrogenation have been recently reviewed [11,21,34]. Furthermore, some reports concerning the use of non-noble metals such as Co [35–36], Ni [37–38], Al [39–40], and Mn [41–43] have been released. In this scenario, copper systems remain widely unexplored and, generally, have displayed low activity, regardless of the oxidation state of the Cu precursor and of the presence/absence of additives [44–47]. Finally, a Cu(I)–formato complex able to promote FA dehydrogenation in the absence of any additive was very recently reported by Berthet, Cantat and co-workers [48].

**Figure 1:**
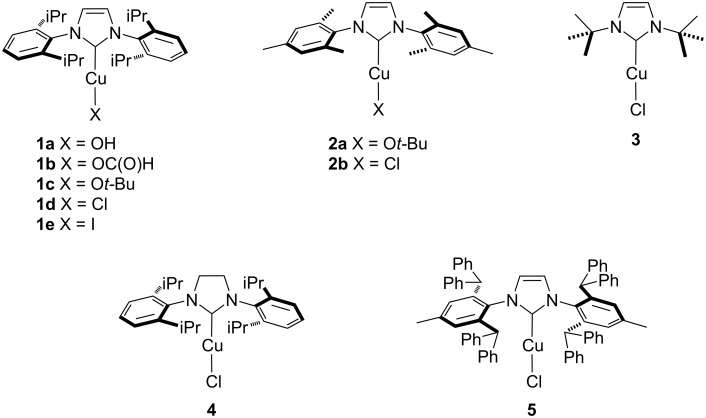
NHC–Cu complexes investigated in this study.

During the past decades, NHC–Cu complexes have shown to be efficient catalysts in a plethora of reactions. Indeed, they guarantee better performances than copper salts, even with lower catalyst loading and under milder conditions [49–51]. Based on this background, the first potential formic acid dehydrogenation catalyzed by NHC–Cu complexes (Figure 1) was investigated.

## Results and Discussion

**Scheme 1:**
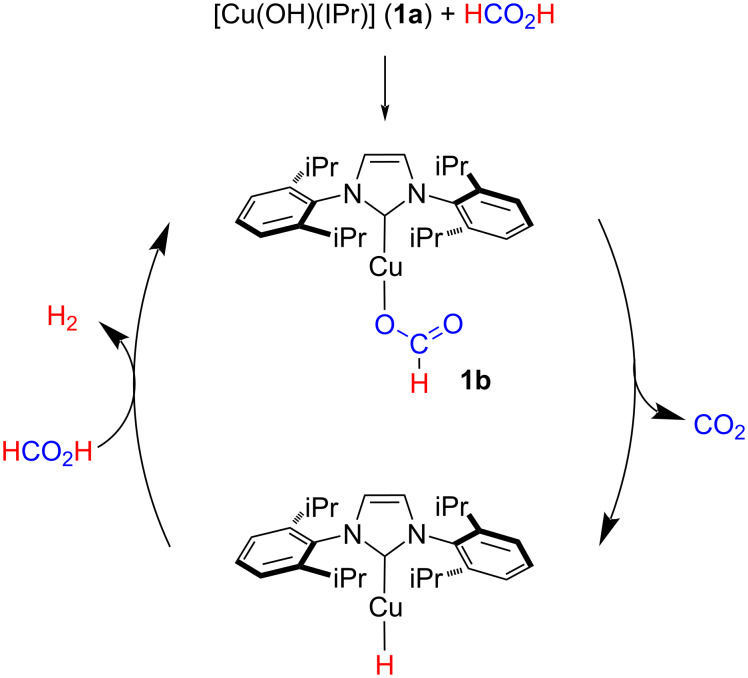
Hypothetical mechanism for FA decomposition via decarboxylation of NHC–Cu–formato species.

We have shown that the reaction of [Cu(OH)(IPr)] (**1a**) with formic acid provides the formato species [Cu{OC(O)H}(IPr)] (**1b**) [52–53]. It was hypothesized that the thermal decarboxylation of **1b** would generate in situ a highly reactive hydride species. The reaction of the latter with formic acid would regenerate the formato complex and release H_2_ (Scheme 1). To support these working hypotheses, the reaction of 1 equiv [Cu(OH)(IPr)] with 2 equiv of formic acid in a sealed tube was followed by ^1^H NMR spectroscopy in deuterated toluene (C_7_D_8_). After 16 h at 110 °C, the formation of **1b** was observed while no excess of formic acid was detected. Interestingly, by adding to the reaction mixture a further equivalent of FA, evolution of gas was observed and H_2_ was detected by NMR spectroscopy (see Supporting Information File 1, Figure S1). This highlighted the formation in situ of the Cu–H species by decarboxylation of **1b**.

**Figure 2:**
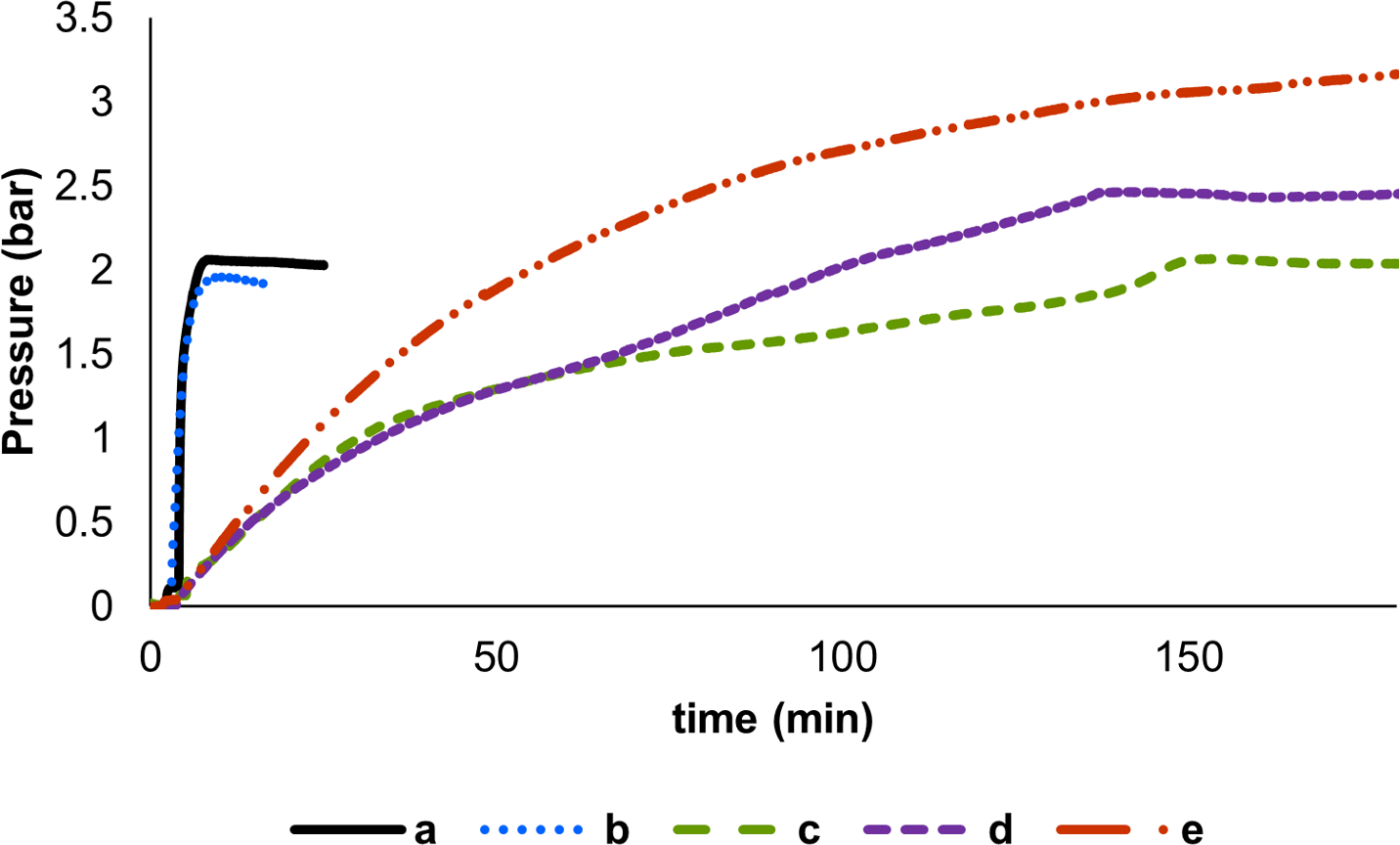
Decomposition of FA catalyzed by NHC–Cu complexes in the presence of PhSiH_3_. Reaction conditions: formic acid (0.5 mmol, 1 equiv), toluene 2 mL, 3 h. **1a** 10 mol %, PhSiH_3_ (1 equiv), 25 °C (a); **1c** 10 mol %, PhSiH_3_ (1 equiv), 25 °C (b); **2a** 10 mol %, PhSiH_3_ (1 equiv), 25 °C (c); **1a** 1 mol %, PhSiH_3_ (1 equiv), 25 °C (d); **1a** 0.1 mol %, PhSiH_3_ (1 equiv), 40 °C (e).

To quantify the amount of gas produced, the pressure change was measured as a function of time (see Supporting Information File 1, Table S1). By heating formic acid in toluene in the presence of 1 and 10 mol % of **1a**, no evolution of gas was observed neither at 25 nor at 110 °C (see Supporting Information File 1, Table S1, entries 1–4); conversely, by increasing the catalyst loading to 30 mol % (with respect to FA), an encouraging 26% conversion was observed at 110 °C (see Supporting Information File 1, Table S1, entry 5). However, to decrease the temperature and the catalyst loading, the generation of the Cu–H species by means of a silane was considered [52–57]. By performing the reaction in the presence of a catalytic amount of PhSiH_3_ (30 mol %, 1:1 ratio with respect to [Cu]), no significant conversion was obtained even at 50 °C with high catalyst loading (see Supporting Information File 1, Table S1, entries 6–8). To our delight, upon reacting in toluene equimolar amounts of FA and PhSiH_3_, efficiency increased (Figure 2). Indeed, by using **1a** and its *tert*-butoxide congener **1c** ([Cu] = 10 mol %) a violent evolution of gas was observed at room temperature, resulting into a considerable increase of pressure which reached a plateau within minutes (Figure 2a and b). Such a value corresponded to the production of ca. 1.5 equivalents of gas (see Supporting Information File 1, section 4).

Notably, when [Cu(O*t*-Bu)(IMes)] (**2a**) was employed, the reaction proceeded slowly (Figure 2c). This can be due to the faster decomposition of its corresponding hydride species with respect to the IPr-based analogues. Remarkably, catalyst loadings as low as 1 mol % with complex **1a** proved to promote the reaction, albeit at a slower rate (Figure 2d). Finally, at 40 °C and with only 0.1 mol % of **1a** and 1 equivalent of PhSiH_3_ (Figure 2e), almost 2 equivalents of gas were produced from 1 equivalent of formic acid within 3 hours.

It has to be noted that, since all reactions were conducted in solution, all copper complexes must be considered as monomeric. In fact, dimeric Cu–H and Cu–formato are generally observed only in the solid state [53,58].

**Figure 3:**
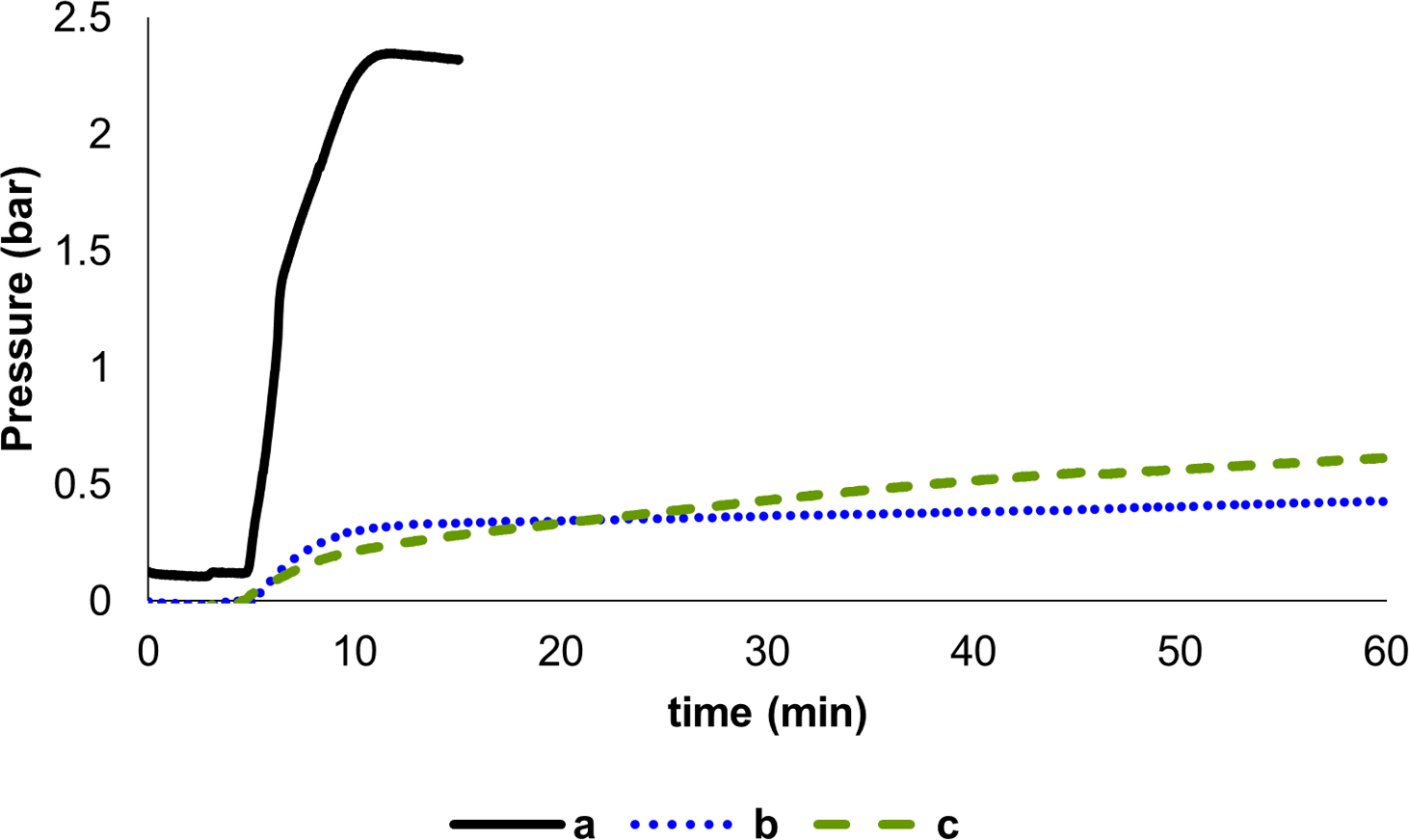
Decomposition of FA catalyzed by NHC–Cu complexes in the presence of different silanes. Reaction conditions: formic acid (0.5 mmol, 1 equiv), silane (1 equiv), toluene 2 mL, **1a** (10 mol %), 25 °C, 1 h. PhSiH_3_ (a); Me(EtO)_2_SiH (b); (EtO)_3_SiH (c).

Other silanes were also investigated (Figure 3). Under the same reaction conditions (toluene, equimolar amounts of FA and silane, 25 °C, 10 mol % of **1a**), both Me(EtO)_2_SiH and (EtO)_3_SiH showed to be ca. 5 times less efficient than PhSiH_3_. The composition of the gas evolved from the FA decomposition in the presence of PhSiH_3_ was investigated. Surprisingly, gas-chromatography analyses showed that only H_2_ was released. Remarkably, neither CO nor CO_2_ were detected (see Supporting Information File 1, section 5).

**Scheme 2:**
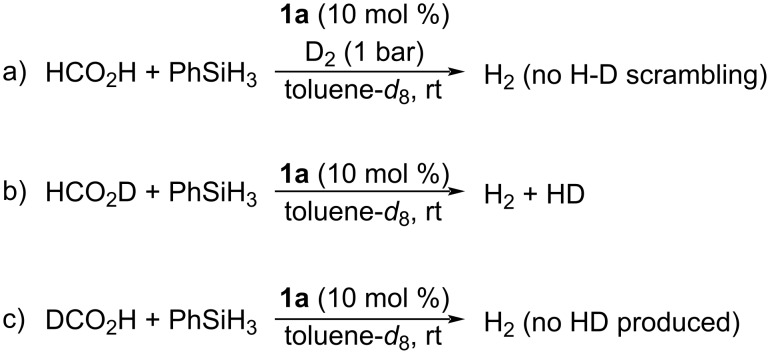
Isotopic labeling experiments.

To obtain more information on the mechanism of this reaction, isotopic labeling experiments were carried out (Scheme 2). By reacting FA and PhSiH_3_ (1:1 molar ratio) in the presence of 10 mol % of **1a**, in deuterated toluene at room temperature under D_2_ atmosphere (1 atm), no H–D scrambling was observed (Scheme 2a). Furthermore, the **1a**-catalyzed reaction of FA labeled at the acidic position with PhSiH_3_ afforded both HD and H_2_ (Scheme 2b, see Supporting Information File 1, Figure S2). Unfortunately, by using DCO_2_H, no HD was produced (Scheme 2c). This last result showed that formic acid does not undergo complete dehydrogenation.

**Scheme 3:**
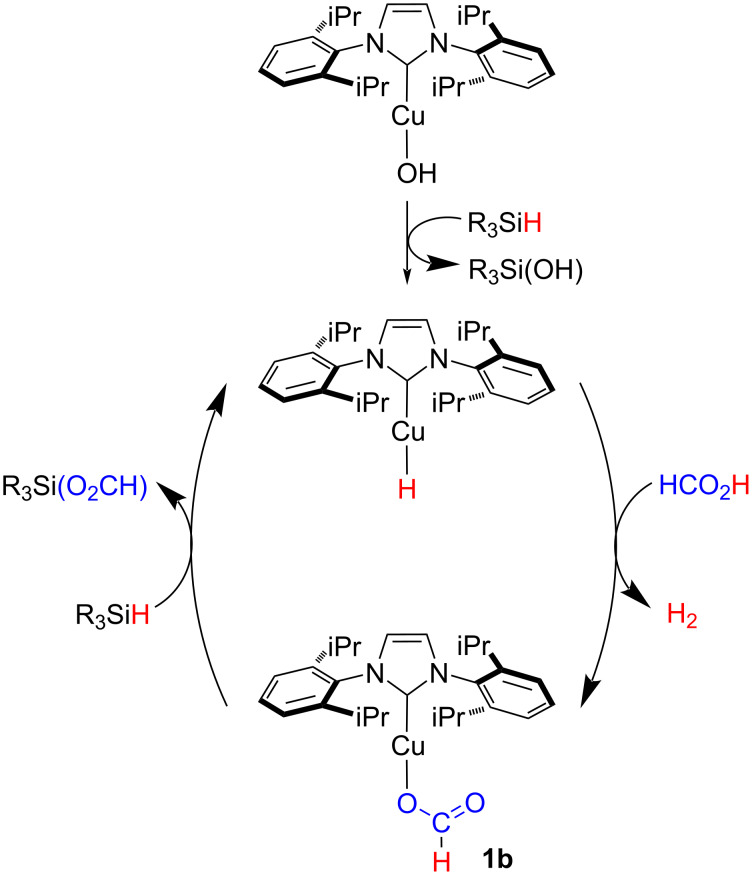
Proposed catalytic cycle for the NHC–Cu-catalyzed FA dehydrogenation.

Hence, it was hypothesized that the production of more than 1 equivalent of hydrogen was due to concurrent mechanisms at play. A proposed catalytic cycle is depicted in Scheme 3. The first step involves the reaction of [Cu(OH)(IPr)] with phenylsilane affording silanol and a highly reactive Cu–H species [53–58]. The latter reacts with FA in an acid–base fashion releasing hydrogen and generating a Cu–formato complex. This species reacts with the silanol released during the first step (or with another hydrosilane present in the reaction medium) regenerating Cu–H. This step would also result into the sequestration of CO_2_ via hydrosilylation affording silyl formate products.

**Scheme 4:**
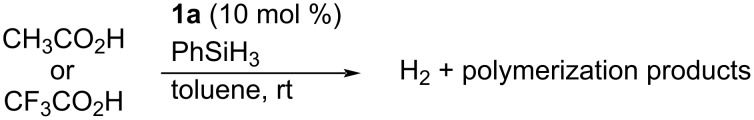
Dehydrogenative coupling of phenylsilane.

Excess H_2_ can be generated by dehydrogenative coupling of those species in the presence of the copper catalyst. Indeed, metal hydride systems have shown high catalytic activity in such a reaction [59–60]. In addition, the formation of products compatible with the polymerization of silicon compounds was observed in all reactions (see Supporting Information File 1, Figure S3). Noteworthy, by replacing formic acid with acetic acid only 1 equivalent of hydrogen was released. The different reactivity towards the dehydrogenative coupling of the silyl carboxylate species can be due to steric reasons rather than electronics. In fact, the reaction of trifluoroacetic acid led to the same outcome as acetic acid (Scheme 4, see Supporting Information File 1, section 8). Theoretical as well as experimental investigations to assess the mechanism of this transformation are still ongoing.

The simultaneous occurrence of two dehydrogenative mechanisms can explain the results of the isotopic labeling experiments. Indeed, while HD arises from the coupling of HCO_2_D with phenylsilane, the formation of H_2_ takes place by dehydrogenative coupling of the hydrosilanes present in the reaction mixture.

To simplify the catalytic system leading to a decomposition pathway in line with previous reports, the replacement of silanes with other additives was considered. It has been shown that the addition of amines may be crucial for the catalytic FA dehydrogenation. Mixtures of FA/triethylamine have been intensively investigated [44,61]. Thus, the decomposition of a 1:1 HCO_2_H/NEt_3_ mixture in the presence of different NHC–Cu complexes was explored (Table 1). No conversion was achieved with [Cu(OH)(IPr)] (**1a**). To our delight, the reaction in the presence of its chloride analogue **1d** led to 24% conversion after 3 hours (Table 1, entry 2). Indeed, this complex proved to be three times more efficient than CuI, the best catalyst identified for this transformation in previous studies [44]. By replacing the chloride ligand with iodine, the conversion drastically decreased (Table 1, entry 4). The same effect was observed by using complexes bearing an NHC ligand with *N*-alkyl substituents or a saturated backbone (Table 1, entries 5 and 6).

**Table 1:** Catalyst optimization.^a^

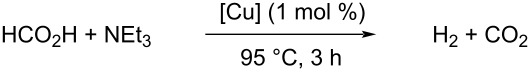						

entry	[Cu]	*V* (mL)^b^	H_2_ (mmol)	conversion (%)	TON	TOF (h^−1^)

1	**1a**	–	–	–	–	–
2	**1d**	300	7	24	27	7
3	CuI	100	2	8	9	3
4	**1e**	55	1	4	4.4	1.5
5	**3**	65	2	5	6	2
6	**4**	50	1	4	4	2
7	**2b**	50	1	4	4.4	1.5
**8**	**5**	**450**	**10**	**36**	**40**	**13**

^a^Reaction conditions: HCO_2_H/NEt_3_ 1:1 (28 mmol), [Cu] 1 mol %, 95 °C, 3 h. ^b^Measured by means of a gas burette.

Finally, the effect of the bulkiness of the ligand was investigated. In the presence of [Cu(Cl)(IPr*)] (**5**) bearing a sterically demanding NHC, 36% conversion was achieved while only 4% was obtained with complex **2b** featuring a smaller ligand (Table 1, entries 7 and 8). Analysis of the gas phase showed that both H_2_ and CO_2_ were released while no CO was detected (see Supporting Information File 1). Remarkably, the TOF measured using **5** was ca. 1 order of magnitude higher than those previously reported for other Cu/amine systems under similar reaction conditions [44–47]. The influence of the nature of the amine was next studied (Table 2). In the absence of the amine, no conversion was observed (Table 2, entry 1). When aniline was used, a slight improvement of the conversion was obtained with respect to the reaction involving triethylamine (Table 2, entries 2 and 3).

**Table 2:** Optimization of the amine.^a^

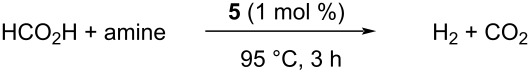			

entry	amine	p*K*_a_	conversion (%)

1	–	–	none
2	aniline	4.58	41
3	NEt_3_	11.1	36
4	ethylenediamine	10.7	26
5	*p*-chloroaniline	3.81	25
6	diphenylamine	13.2	19
7	diethylamine	11.0	16
8	*p*-anisidine	5.29	16
9	pyridine	5.25	14
10	*p*-toluidine	5.07	13
11	piperidine	11.1	13

^a^Reaction conditions: HCO_2_H (3.5 mmol, 1 equiv), amine (3.5 mmol, 1 equiv), **5** (1 mol %), 95 °C, 3 h.

Upon employing ethylenediamine, the conversion dropped to 26% (Table 2, entry 4). This may be due to the chelating behavior of this amine. Indeed, ethylenediamine can strongly coordinate the metal center generating an inactive species [44]. Electronic effects were next investigated. Electron-rich anilines like *p*-toluidine or *p*-anisidine led to lower conversions than *p*-chloroaniline (Table 2, entries 5, 8, and 10). As highlighted in Table 2, no correlation between the basicity of the amines and the dehydrogenation of formic acid was found. This is in agreement with the literature [44]. Interestingly, an almost linear correlation between the FA conversion and the cone angle (Θ) of the alkylamines was found (Table 3) [62].

**Table 3:** Influence of the size of the amine on the dehydrogenation of FA/amine mixtures.

amine	cone angle Θ (°) [62]	conversion (%)

NEt_3_	150	36
diphenylamine	136	19
diethylamine	125	16
piperidine	120	13

Remarkably, higher conversions in the presence of bulky amines were reported also for other catalytic systems [16–17,44]. It is important to note that aniline, albeit being less sterically demanding than other amines (Θ = 111°), importantly deviates from this trend, allegedly because of its aromaticity. It is reasonable to assume that the role of the amine is not only limited to the deprotonation of the acid; indeed, their coordination to the copper center must also be taken into account [45]. Moreover, the reaction between FA and amines occurs in a more complex manner than a common acid–base equilibrium. In fact, calorimetric studies on the formation of FA/triethylamine adducts showed that the reaction mixture is composed of more than one species presenting different ammonium/formato ratios [63].

Compared to other Cu-based catalysts employed in FA dehydrogenation, the complex **5**/aniline system exhibited higher activity (TOF 14 h^−1^) than monomeric formate species bearing bulky phenanthroline ligands (TOF 4 h^−1^) [48] as well as Cu(0), Cu(I), and Cu(II) precursors/NEt_3_ (TOFs < 1 h^−1^) [44]. In contrast, [Cu(NCMe)_4_][PF_6_]/phosphine ligands/(*t*-BuNC–NEt_3_) systems represent the most active Cu-based catalysts reported to date for this transformation, exhibiting TOFs spanning from 10 to 240 h^−1^ [46]. Nevertheless, these systems still show inferior performance compared to Fe [33], Ru [15], and Ir [28], allowing for TOFs of up to 10^5^ h^−1^.

## Conclusion

The production of hydrogen from formic acid catalyzed by NHC–Cu complexes was investigated. The reaction in the presence of PhSiH_3_ led to the production of an over-stoichiometric amount of hydrogen deriving from the existence of two dehydrogenative pathways while CO_2_ was sequestrated into silyl formate species. In addition, NHC–Cu complexes proved more efficient than copper salts investigated in previous studies in the dehydrogenation of the conventional system FA/amine. Synthetic and theoretical studies are still ongoing in our laboratories to obtain more insights into the mechanism of this reaction.

## Supporting Information

File 1 Detailed description of the procedure for the gas evolution experiments, NMR spectra of isotopic labeling experiments.

## Data Availability

All data that supports the findings of this study is available in the published article and/or the supporting information of this article.
