# Supplementary material for: Hydrogen production from formic acid catalyzed by NHC–Cu complexes
Source: Beilstein J Org Chem. 2026 Apr 23;22:620–7. doi: 10.3762/bjoc.22.48 (PMC13159329; doi:10.3762/bjoc.22.48)
Supplement: File 1 — Detailed description of the procedure for the gas evolution experiments, NMR spectra of isotopic labeling experiments. [file Beilstein_J_Org_Chem-22-620-s001.pdf]

BEILSTEIN JOURNAL OF ORGANIC CHEMISTRY

Supporting Information

for

Hydrogen production from formic acid catalyzed by NHC–Cu complexes

Orlando Santoro and Catherine S. J. Cazin

Beilstein J. Org. Chem. **2026,** *22,* 620–627. doi:10.3762/bjoc.22.48

Detailed description of the procedure for the gas evolution experiments, NMR spectra of isotopic labeling experiments

License and Terms: This is a supporting information file under the terms of the Creative Commons Attribution License (https://creativecommons.org/licenses/by/4.0). Please note that the reuse, redistribution and reproduction in particular requires that the author(s) and source are credited and that individual graphics may be subject to special legal provisions.
The license is subject to the *Beilstein Journal of Organic Chemistry* terms and conditions: (https://www.beilstein-journals.org/bjoc/terms)

Table of contents

1. General information....S2
2. Synthesis of [Cu{OC(O)H}(IPr)] (**1b**)[1c,2] S2
3. Preliminary tests S3
   3.1 Formic acid decomposition via decarboxylation of [Cu{OC(O)H}(IPr)] (**1b**) S3
   3.2 Quantification of the amount of gas produced....S3
4. General procedure for the decomposition of formic acid in the presence of silanes (Figures 2 and 3) S4
5. Procedure for the determination of the gas composition....S4
6. General procedure for the isotopic labeling NMR experiments....S5
   6.1 Decomposition of HCO_2D S5
   6.2 Decomposition of DCO_2H S6
   6.3 Decomposition of HCO_2H under D_2 atmosphere S6
7. Analysis of the reaction mixture of the formic acid dehydrogenation in the presence of $PhSiH_3$ S6
8. Dehydrogenative coupling of phenylsilane with acetic acid and TFA....S7
9. Hydrogen production from formic acid/amine mixtures. S8
   9.1 Optimization of the catalyst....S8
   9.2 Optimization of the amine S8
10. ^{1}H and $^{13}C\{^{1}H\}$ NMR spectra of [Cu{OC(O)H}(IPr)] (**1b**)....S9
11. References....S10

1. General information

All reactions were carried out under argon atmosphere using standard Schlenk and glovebox techniques unless otherwise stated. NHC–Cu(I) complexes were synthesized following reported procedures.[1] Chemicals were used as received unless otherwise stated. Formic acid was purchased form Sigma Aldrich and distilled prior to use. Dry toluene was obtained from a PureSolv SPS-400-5 solvent purification system. ^{1}H and ^{13}C{^{1}H} nuclear magnetic resonance (NMR) spectra were recorded on a Bruker-400 MHz or 300 MHz spectrometer using the residual solvent peak as reference ($CDCl_3$: δ_H = 7.26 ppm, δ_C = 77.16 ppm, CD_2Cl_2: δ_H = 5.32 ppm, δ_C = 53.84 ppm, C_7D_8: δ_H = 2.08 ppm) at 298 K.

Elemental analyses were performed at London Metropolitan University 166-220, Holloway Road, London, N7 8DB.

2. Synthesis of [Cu{OC(O)H}(IPr)] (1b)[1c,2]

$$\text{[Cu(OH)(IPr)]} + HCO_2H \xrightarrow[\text{rt, 1h}]{\text{benzene}} \text{[Cu\{OC(O)H\}(IPr)]} + H_2O \quad 82\%$$

Complex **1b** was obtained as previously described[2] and its characterization data were in agreement with reported analyses.

3. Preliminary tests

3.1 Formic acid decomposition via decarboxylation of [Cu{OC(O)H}(IPr)] (1b)

Under argon atmosphere, a Young NMR tube was charged with [Cu(OH)(IPr)] (**1a**, 23.4 mg, 0.05 mmol, 1 equiv), formic acid (4 µL, 0.1 mmol, 2 equiv), and toluene-d_8. The tube was heated at 110 °C for 16 hours by means of an oil bath. The ^{1}H NMR spectrum of the mixture was then recorded. The formation of [Cu{OC(O)H}(IPr)] (**1b**) was observed while no excess of formic acid was detected (Figure S1, A). Under argon atmosphere, formic acid (2 µL, 0.05 mmol, 1 equiv) was added to the reaction mixture and a violent bubbling was immediately observed. The ^{1}H NMR spectrum showed the formation of H_2 (Figure S1, B).

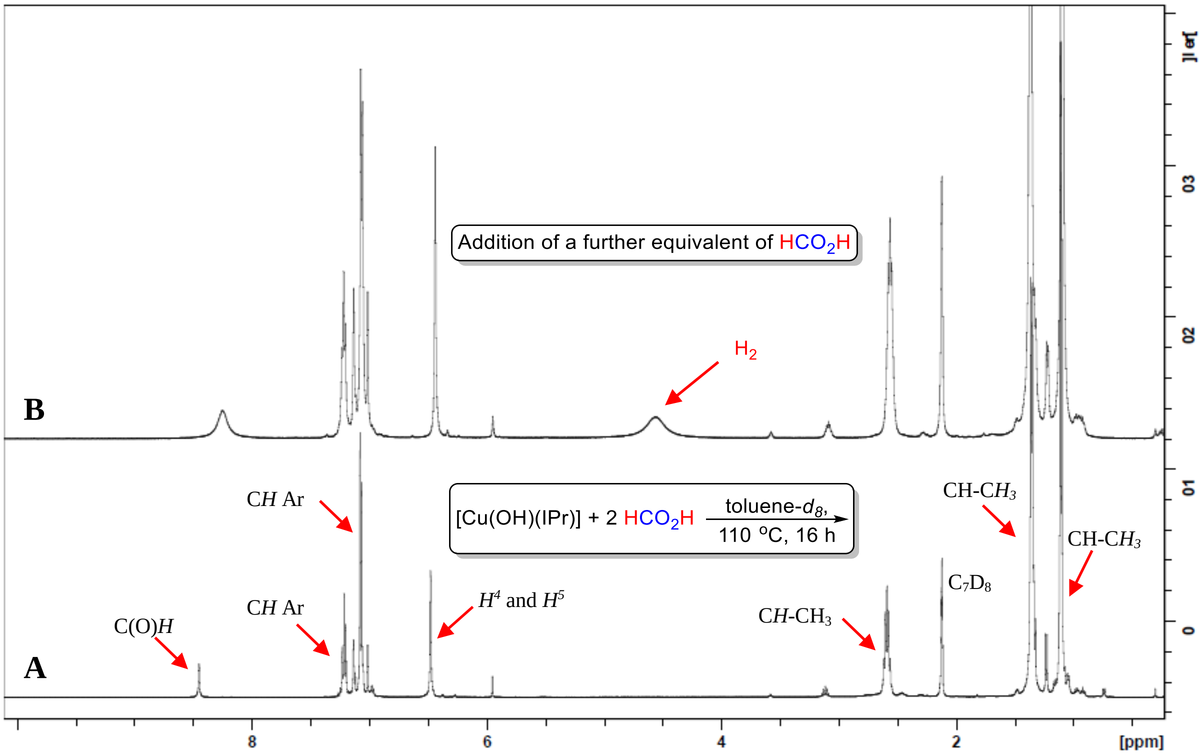

Figure S1 Reactivity of [Cu(OH)(IPr)] (**1a**) with formic acid in C_7D_8. (**A**) Formation of [Cu{OC(O)H}(IPr)] (**1b**). **(B)** Addition of further equivalent of FA to the mixture. H_2 was detected. (H^4 and H^5 = protons of the NHC-ligand backbone)

3.2 Quantification of the amount of gas produced

Under argon atmosphere, a reaction vessel equipped with a magnetic stirring bar was charged with the copper complex and toluene (2 mL). The vessel was closed with a septum cap and connected to a pressure transducer. The system was then allowed to reach the reaction temperature by means of an oil bath. Formic acid (20 µL, 0.5 mmol, 1 equiv) and $PhSiH_3$ (when present) were injected and the change of the pressure as function of time was measured by

using “Man on the moon, Series 101 kit”.[3] The ideal gas law equation $p\,V = n\,R\,T$ was used to calculate the number of moles of H_2 produced.

Table S1 NHC–Cu-catalyzed formic acid decomposition.

$$HCO_2H \xrightarrow[\text{toluene, 3 h}]{\text{[Cu] (x mol\%)}} H_2 + CO_2$$

Entry	[Cu] (mol %)	*T* (°C)	silane (mol %)	conversion[a]
1	[Cu(OH)(IPr)] **1a** (1)	25	–	–
2	[Cu(OH)(IPr)] **1a** (1)	110	–	–
3	[Cu(OH)(IPr)] **1a** (10)	25	–	–
4	[Cu(OH)(IPr)] **1a** (10)	110	–	–
**5**	**[Cu(OH)(IPr)] 1a (30)**	**110**	**–**	**26%**
6	[Cu(O*t*-Bu)(IPr)] **1c** (30)	25	$PhSiH_3$ (30)	<5%
7	[Cu(O*t*-Bu)(IPr)] **1c** (30)	50	$PhSiH_3$ (30)	<5%
8	[Cu(O*t*-Bu)(IPr)] **1c** (10)	25	$PhSiH_3$ (10)	–

[a] $n(H_2)$ were calculated by means of the ideal gas law equation $p\,V = n\,R\,T$ and assuming the formation of an equimolar amount of CO_2 and H_2

4. General procedure for the decomposition of formic acid in the presence of silanes (Figures 2 and 3)

Under argon atmosphere, a reaction vessel equipped with a magnetic stirring bar was charged with the copper catalyst and toluene (2 mL). The vessel was closed with a septum cap and connected to a pressure transducer. The system was then allowed to reach the reaction temperature by means of an oil bath. Formic acid (20 µL, 0.5 mmol, 1 equiv) and the silane (1 equiv) were then injected and the change of the pressure as function of time was measured by using “Man on the moon, Series 101 kit”.[3]

5. Procedure for the determination of the gas composition

Gas samples were collected into gas bags and analyzed by using an Agilent 7890 GC configured for refinery gas analysis. Hydrogen, carbon monoxide, and carbon dioxide were

separated on molecular sieve 5 Å columns and quantified through a thermal conductivity detector. A certificated gas mixture, refinery test gas sample (Scott Specialty Gases), was used for calibration purposes.

6. General procedure for the isotopic labeling NMR experiments

Under argon atmosphere, a Young NMR tube was charged with [Cu(OH)(IPr)] (**1a**, 11.2 mg, 10 mol %), C_7D_8 (0.5 mL), labeled formic acid (10.1 µL, 0.26 mmol, 1.05 equiv), and $PhSiH_3$ (33 µL. 0.25 mmol, 1 equiv). The tube was sealed and shaken for 5 minutes at room temperature. The mixture was analyzed by ^1H NMR spectroscopy.

6.1 Decomposition of HCO_2D

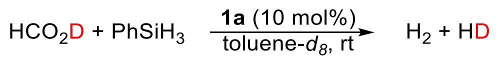

By following the general procedure, the decomposition of HCO_2D (10.1 µL, 0.26 mmol, 1.05 equiv) led to the evolution of both H_2 and HD (Figure S2).

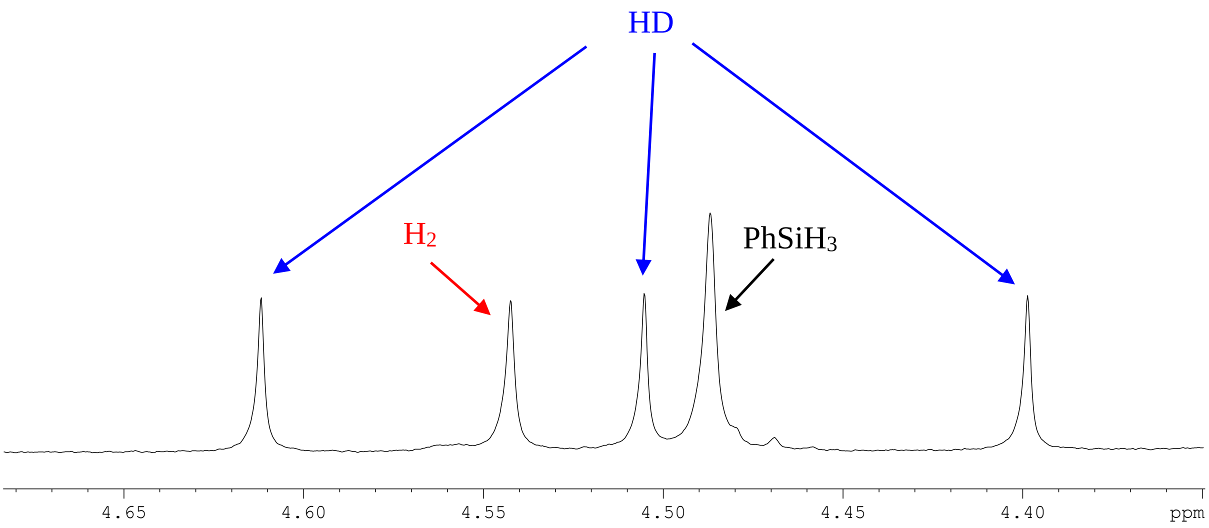

Figure S2 Evolution of H_2 and HD from the Cu-catalyzed decomposition of HCO_2D in the presence of $PhSiH_3$ (^1H NMR in C_7D_8).

6.2 Decomposition of DCO_2H

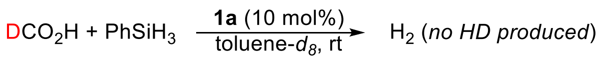

By following the general procedure, the decomposition of DCO_2H (10.1 µL, 0.26 mmol, 1.05 equiv) led to the evolution of H_2.

6.3 Decomposition of HCO_2H under D_2 atmosphere

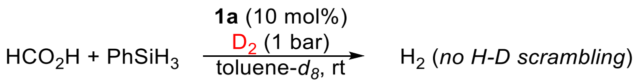

Similar to the general procedure, a Young NMR tube was charged with [Cu(OH)(IPr)] (**1a**, 11.2 mg, 10 mol %), C_7D_8 (0.5 mL), and HCO_2H (10 µL, 0.25 mmol, 1 equiv) under argon atmosphere. The system was evacuated and $PhSiH_3$ (33 µL. 0.25 mmol, 1 equiv) was added. After a further evacuation, D_2 was flushed into the system for 5 minutes. Only H_2 was detected on the ^{1}H NMR spectrum.

7. Analysis of the reaction mixture of the formic acid dehydrogenation in the presence of $PhSiH_3$

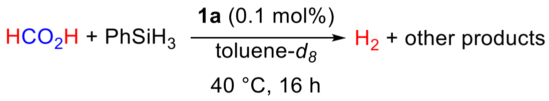

Under argon atmosphere, a Young NMR tube was charged with a stock solution of [Cu(OH)(IPr)] (**1a**) in CH_2Cl_2 (50 µL, corresponding to 0.1 mol % [Cu]). The solvent was then removed under reduced pressure. Thereafter, C_7D_8 (0.5 mL), formic acid (20 µL, 0.5 mmol, 1.0 equiv) and $PhSiH_3$ (66 µL. 0.5 mmol, 1 equiv) were added. The tube was sealed and heated at 40 °C for 16 hours by means of an oil bath. The mixture was analyzed by $^{13}C\{^{1}H\}$ NMR spectroscopy (Figure S3).

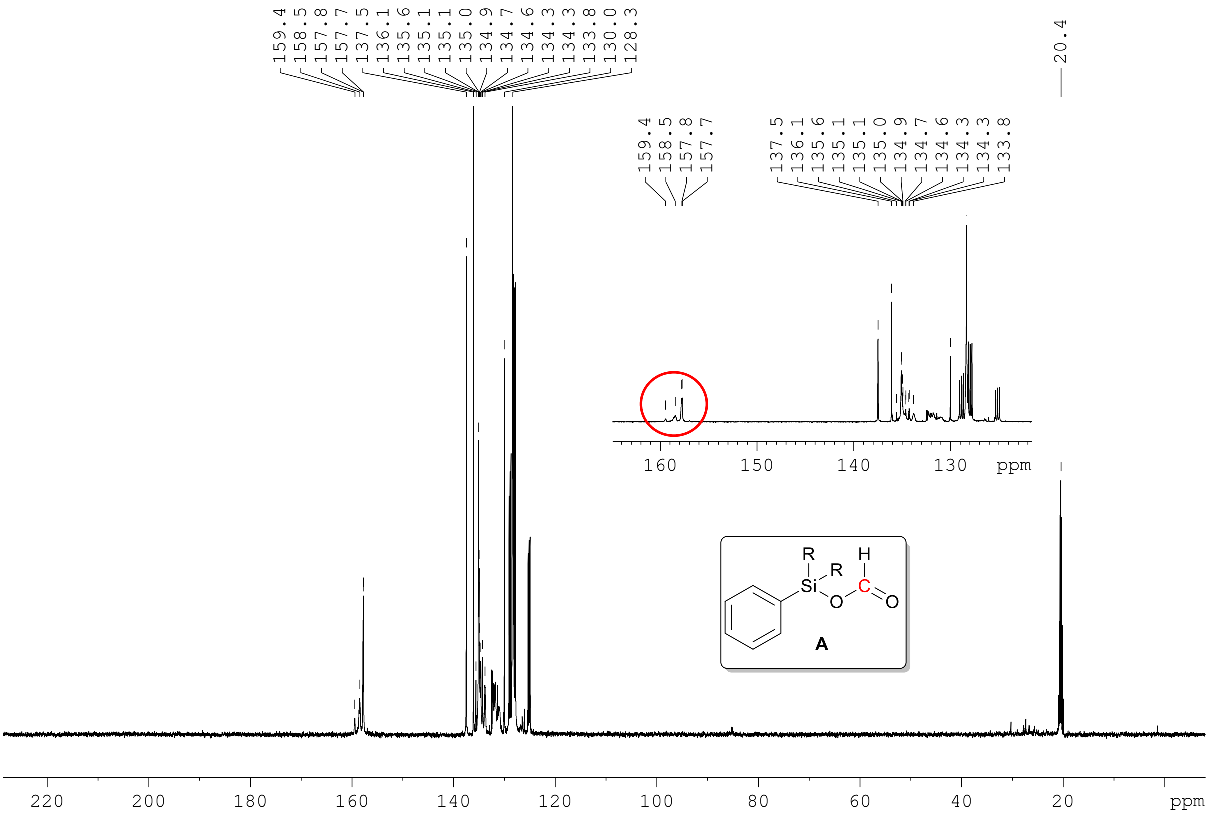

Figure S3: $^{13}C\{^{1}H\}$ NMR spectrum (in C_7D_8) of the reaction mixture from the Cu-catalyzed formic acid decomposition. The signals in the red circle are compatible with the formation of silyl-fomate products (**A**).

The non-selective spectrum highlighted the plausible polymerization of silicon-based compounds. In addition, the signals at ≈160 ppm are compatible with the formation of $PhSiH_{3n}(O_2CH)_n$ products.[4]

8. Dehydrogenative coupling of phenylsilane with acetic acid and TFA

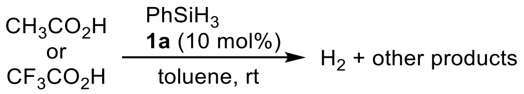

Under argon atmosphere, a reaction vessel equipped with a magnetic stirring bar was charged with [Cu(OH)(IPr)] (**1a**, 23.4 mg, 0.05mmol, 10 mol %) and toluene (2 mL). The vessel was closed with a septum cap and connected to a pressure transducer. Acetic acid or trifluoroacetic acid (0.5 mmol, 1 equiv) and phenylsilane (62 µL, 0.5 mmol, 1 equiv) were then injected. The mixture was stirred at room temperature until the pressure (measured by using "Man on the moon, Series 101 kit")[3] reached a constant value.

9. Hydrogen production from formic acid/amine mixtures.

9.1 Optimization of the catalyst

A mixture of formic acid (1.1 mL, 28 mmol, 1 equiv) and triethylamine (3.9 mL, 28 mmol, 1 equiv) was pre-heated to 95 °C (oil bath) in a 25 mL two-necked flask equipped with a reflux condenser which was connected to a gas burette. The system was purged with N_2 prior to the start of the reaction by adding the solid copper catalyst (0.28 mmol, 1 mol %). The volume of gas produced was measured by means of the gas burette.

9.2 Optimization of the amine

A mixture of HCO_2H (0.14 mmol, 1 equiv) and the amine (3.5 mmol, 1 equiv), was pre-heated at 95 °C in a 5 mL flask equipped with a reflux condenser which was connected to a gas burette. The system was purged with N_2 prior to start the reaction by adding solid [Cu(Cl)(IPr*)] (**5**, 33 mg, 1 mol %), 95 °C, 3 h. The volume of gas produced was measured by means of the gas-burette.

10. ^{1}H and $^{13}C\{^{1}H\}$ NMR spectra of [Cu{OC(O)H}(IPr)] (1b)

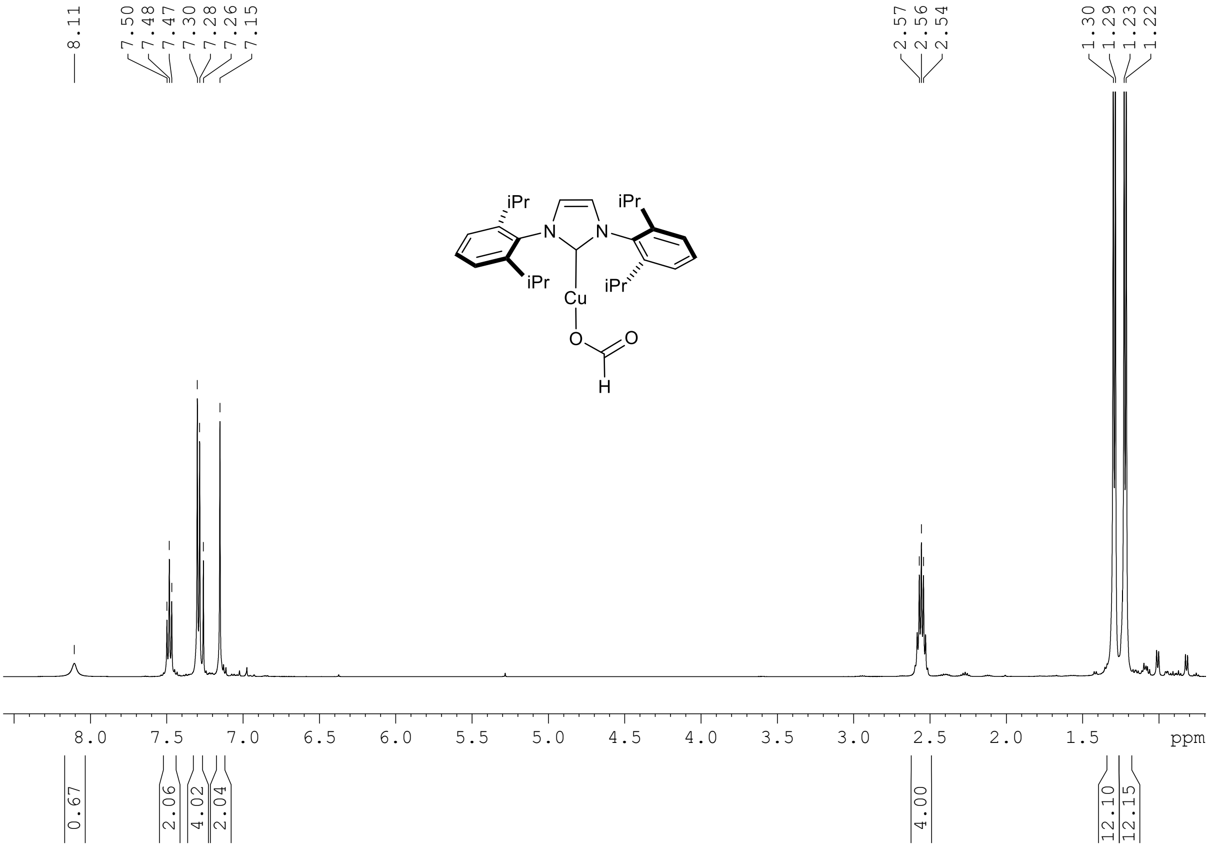

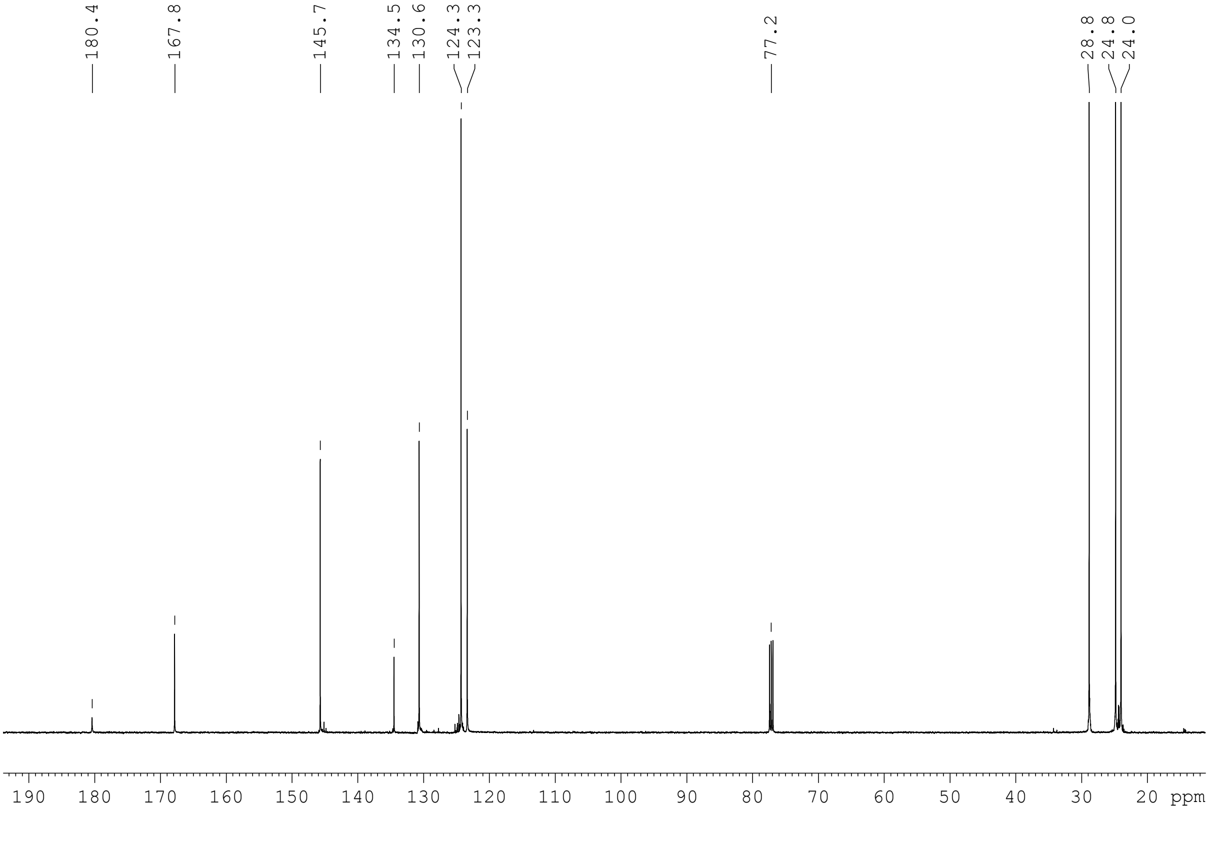

11. References

1. (a) O. Santoro, A. Collado, A. M. Z. Slawin, S. P. Nolan and C. S. J. Cazin, *Chem. Commun.*, 2013, **49**, 10483. (b) G. C. Fortman, A. M. Z Slawin and S. P. Nolan, *Organometallics*, 2010, **29**, 3966. (c) L. Zhang, J. Cheng, and Z. Hou, *Chem. Commun*., 2013, **49**, 4782. (d) K. Motokura, D. Kashiwame, N. Takahashi, A. Miyaji and T. Baba, *Chem. Eur. J.*, 2013, **19**, 10030.
2. O. Santoro, F. Lazreg, Y. Minenkov, L. Cavallo and C. S. J. Cazin, *Dalton Trans.*, 2015, **44**, 18138.
3. Kit designed to monitor the progress of reactions evolving gases by measuring the pressure increase vs. time in closed reaction systems. https://manonthemoontech.com/kinetics-kits/x104-gas-evolution/
4. P. Steinhoff, M. Paul, J. P. Schroers and M. E. Tauchert, *Dalton Trans.*, 2019, **48**, 1017.
